# A Virtual Look at Epstein–Barr Virus Infection: Biological Interpretations

**DOI:** 10.1371/journal.ppat.0030137

**Published:** 2007-10-19

**Authors:** Karen A Duca, Michael Shapiro, Edgar Delgado-Eckert, Vey Hadinoto, Abdul S Jarrah, Reinhard Laubenbacher, Kichol Lee, Katherine Luzuriaga, Nicholas F Polys, David A Thorley-Lawson

**Affiliations:** 1 Virginia Bioinformatics Institute, Virginia Polytechnic and State University, Blacksburg, Virginia, United States of America; 2 Department of Biochemistry and Biotechnology, Kwame Nkrumah University of Science and Technology, Kumasi, Ghanna, West Africa; 3 Department of Pathology, Tufts University School of Medicine, Boston, Massachusetts, United States of America; 4 Zentrum Mathematik der Technischen Universität, München, Garching bei München, Germany; 5 Pediatrics and Molecular Medicine, University of Massachusetts Medical School, Worcester, Massachusetts, United States of America; 6 Research and Cluster Computing, Virginia Tech Information Technology, Virginia Polytechnic and State University, Blacksburg, Virginia, United States of America; University of Wisconsin-Madison, United States of America

## Abstract

The possibility of using computer simulation and mathematical modeling to gain insight into biological and other complex systems is receiving increased attention. However, it is as yet unclear to what extent these techniques will provide useful biological insights or even what the best approach is. Epstein–Barr virus (EBV) provides a good candidate to address these issues. It persistently infects most humans and is associated with several important diseases. In addition, a detailed biological model has been developed that provides an intricate understanding of EBV infection in the naturally infected human host and accounts for most of the virus' diverse and peculiar properties. We have developed an agent-based computer model/simulation (PathSim, Pathogen Simulation) of this biological model. The simulation is performed on a virtual grid that represents the anatomy of the tonsils of the nasopharyngeal cavity (Waldeyer ring) and the peripheral circulation—the sites of EBV infection and persistence. The simulation is presented via a user friendly visual interface and reproduces quantitative and qualitative aspects of acute and persistent EBV infection. The simulation also had predictive power in validation experiments involving certain aspects of viral infection dynamics. Moreover, it allows us to identify switch points in the infection process that direct the disease course towards the end points of persistence, clearance, or death. Lastly, we were able to identify parameter sets that reproduced aspects of EBV-associated diseases. These investigations indicate that such simulations, combined with laboratory and clinical studies and animal models, will provide a powerful approach to investigating and controlling EBV infection, including the design of targeted anti-viral therapies.

## Author Summary

The possibility of using computer simulation and mathematical modeling to gain insight into biological systems is receiving increased attention. However, it is as yet unclear to what extent these techniques will provide useful biological insights or even what the best approach is. Epstein–Barr virus (EBV) provides a good candidate to address these issues. It persistently infects most humans and is associated with several important diseases, including cancer. We have developed an agent-based computer model/simulation (PathSim, Pathogen Simulation) of EBV infection. The simulation is performed on a virtual grid that represents the anatomy where EBV infects and persists. The simulation is presented on a computer screen in a form that resembles a computer game. This makes it readily accessible to investigators who are not well versed in computer technology. The simulation allows us to identify switch points in the infection process that direct the disease course towards the end points of persistence, clearance, or death, and identify conditions that reproduce aspects of EBV-associated diseases. Such simulations, combined with laboratory and clinical studies and animal models, provide a powerful approach to investigating and controlling EBV infection, including the design of targeted anti-viral therapies.

## Introduction

Computer simulation and mathematical modeling are receiving increased attention as alternative approaches for providing insight into biological and other complex systems [1]. An important potential area of application is microbial pathogenesis, particularly in cases of human diseases for which applicable animal models are lacking. To date, most simulations of viral pathogenesis have tended to focus on HIV [2–7], and employ mathematical models based on differential equations. None have addressed the issue of acute infection by the pathogenic human herpes virus Epstein–Barr virus (EBV) and its resolution into lifetime persistence. With the ever-increasing power of computers to simulate larger and more complex systems, the possibility arises of creating an *in silico* virtual environment in which to study infection. We have used EBV to investigate the utility of this approach. EBV is a human pathogen, associated with neoplastic disease, that is a paradigm for understanding persistent infection in vivo and for which a readily applicable animal model is lacking (reviewed in [8,9]). Equally important is that EBV infection occurs in the lymphoid system, which makes it relatively tractable for experimental analysis and has allowed the construction of a biological model of viral persistence that accounts for most of the unique and peculiar properties of the virus [10,11]. We are therefore in a position to map this biological model onto a computer simulation and then ask how accurately it represents EBV infection (i.e., use our knowledge of EBV to test the validity of the simulation) and whether the matching of biological observation and simulation output provides novel insights into the mechanism of EBV infection. Specifically, we can ask if it is possible to identify critical switch points in the course of the disease where small changes in behavior have dramatic effects on the outcome. Examples of this would be the switch from clinically silent to clinically apparent infection and from benign persistence to fatal infection (as occurs in fatal acute EBV infection and the disease X-linked lymphoproliferative [12], for example), or to clearance of the virus. Indeed, is clearance ever possible, or do all infections lead inevitably to either persistence or death? Such an analysis would be invaluable. Not only would it provide insight into the host–virus balance that allows persistent infection, but it would also reveal the feasibility and best approaches for developing therapeutic interventions to diminish the clinical symptoms of acute infection, prevent fatal infection, and/or clear the virus.

**Figure 1:**
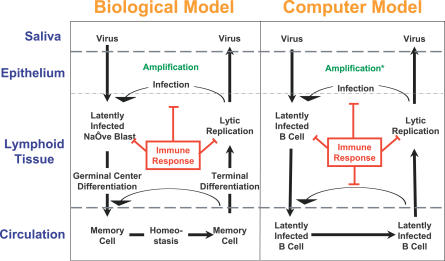
Diagrammatic Representation of the Simulation and the Biological Model on which It Is Based For a detailed description of the biological model, see Introduction and [9–11]. The simulation reflects the biological model with some simplifications (for details see Methods). * denotes that amplification in the simulation is achieved by arbitrarily adding more Vir to the epithelial cell layer at each time step.

A diagrammatic version of the biological model is presented in Figure 1. EBV enters through the mucosal surface of the Waldeyer ring, which consists of the nasopharyngeal tonsil (adenoids), the paired tubal tonsils, the paired palatine tonsils, and the lingual tonsil arranged in a circular orientation around the walls of the throat. Here EBV infects and is amplified in epithelium. It then infects naïve B cells in the underlying lymphoid tissue. The components of the ring are all equally infected by the virus [13]. EBV uses a series of distinct latent gene transcription programs, which mimic a normal B cell response to antigen, to drive the differentiation of the newly infected B cells. During this stage, the infected cells are vulnerable to attack by cytotoxic T cells (CTLs) [14]. Eventually, the latently infected B cells enter the peripheral circulation, the site of viral persistence, as resting memory cells that express no viral proteins [15] and so are invisible to the immune response. The latently infected memory cells circulate between the periphery and the lymphoid tissue [13]. When they return to the Waldeyer ring they are occasionally triggered to terminally differentiate into plasma cells. This is the signal for the virus to begin replication [16], making the cells vulnerable to CTL attack again [14]. Newly released virions may infect new B cells or be shed into saliva to infect new hosts, but are also the target of neutralizing antibody.

Primary EBV infection in adults and adolescents is usually symptomatic and referred to as infectious mononucleosis (AIM). It is associated with an initial acute phase in which a large fraction (up to 50%) of circulating memory B cells may be latently infected [17]. This induces the broad T lymphocyte immune response characteristic of acute EBV infection. Curiously, primary infection early in life is usually asymptomatic. In immunocompetent hosts, infection resolves over a period of months into a lifelong persistent phase in which ∼1 in 10^5^ B cells carry the virus [18]. Exactly how persistent infection is sustained is unclear. For example, once persistence is established, it is unknown if the pool of latently infected memory B cells is self-perpetuating or if a low level of new infection is necessary to maintain it. Indeed, we do not know for sure that the pool of latently infected B cells in the peripheral memory compartment is essential for lifetime persistence. It is even unclear whether the virus actually establishes a steady state during persistence or continues to decay, albeit at an ever slower rate [17].

In the current study we describe the creation and testing of a computer simulation (PathSim) that recapitulates essential features of EBV infection. The simulation has predictive power and has utility for experiment design and understanding EBV infection. One practical limitation of available simulation and modeling approaches has been their inaccessibility to the working biologist. This is often due to the use of relatively unfamiliar computer interfaces and output formats. To address these issues, we have presented the simulation via a user-friendly visual interface on a standard computer monitor. This allows the simulation to be launched and output to be accessed and analyzed in a visual way that is simple and easily comprehensible to the non-specialist.

## Results

### An Agent-Based Computer Simulation of EBV Infection: Pathogen Simulation (PathSim)

**Figure 2:**
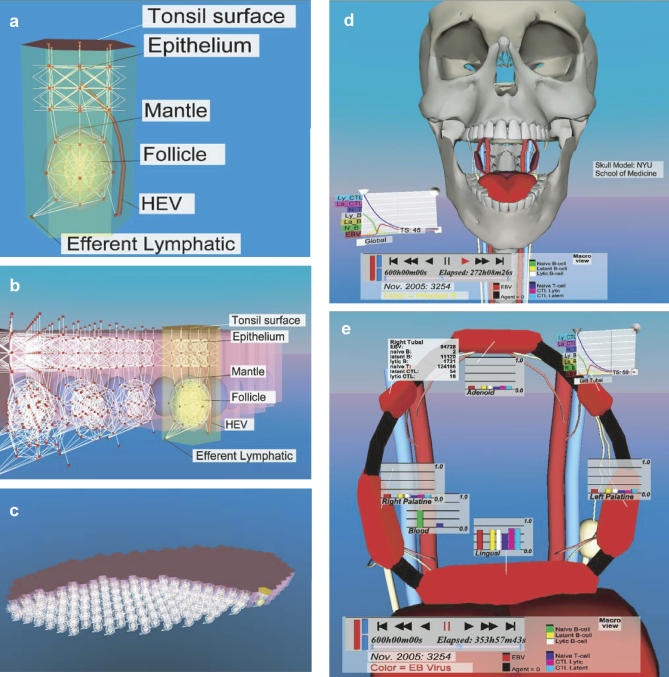
Representative Fields from the Simulation's Virtual Environment (IRVE) For more details on (A–C), see Video S1, and for more on (D and E) see Video S2. (A) The basic unit, used to construct the tonsil grid, consists of a solid hexagonal structure representing surface epithelium, lymphoid tissue, and a single germinal center/follicle. The virtual grid along which the agents move is shown as white lines, and the nodes where agents reside and interact are depicted as red boxes. Each hexagonal unit has one HEV entry point from the peripheral blood and one exit point into the lymphatic system. (B) A view from the side of a single tonsil showing how the hexagonal base units are packed to create the entire grid of the tonsil lymphoepithelium. (C) An overview of a single complete tubal tonsil showing part of its surface and part of its internal structure. (D) Skull level view of the simulation showing the on screen control panel (bottom of figure) and an example of one form of graphic representation of the global status of the infection (line graphs, left). (E) A view at the level of the entire Waldeyer ring demonstrating different methods of quantitation. Graphics in the form of numbers (top left) or bar graphs for a given time step and time line graphs (top right) are shown for various compartments of the simulation. The color signature for the ring is red, which denotes free Vir. The color of the ring denotes the type and level of agent being shown. In this case, the intense red color indicates a high level of free Vir throughout the ring (for an example of infection spreading throughout a single lymph node or the entire ring visualized by changing color intensity, see Figure 3 and Video S2). Note also the draining lymphatics (white vessels) and peripheral circulation (red and blue vessels). These images are screen shots taken on 7/30/2006.

The computer model (PathSim) is a representation of the biological model described in the Introduction. A schematic version of both is shown in Figure 1. To simulate EBV infection, we created a virtual environment consisting of a grid that describes a biologically meaningful topography, in this case the Waldeyer ring (five tonsils and adenoids) and the peripheral circulation, which are the main sites of EBV infection and persistence. The tonsils and adenoids were composed of solid hexagonal base units representing surface epithelium, lymphoid tissue, and a single germinal center/follicle (Figure 2A–2C; Video S1). Each hexagonal unit had one high endothelial venule (HEV) entry point from the peripheral blood and one exit point into the lymphatic system (Figure 2A).

**Figure 3:**
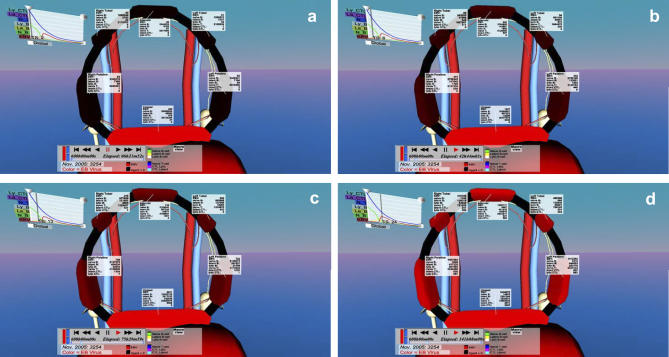
Progression of the Infection in a Typical Simulation as Visualized by the IRVE This Figure is similar to Figure 2E except that different time points in the infection process are shown. Vir were seeded evenly over the lingual tonsil and then allowed to spread to the rest of the ring. Note that in this case as time progresses (A–D) the infection spreads evenly to all of the other tonsils and adenoids as indicated by a gradual uniform increase of color intensity. These images are screen shots taken on 3/7/2007.

Discrete agents (cells or viruses) reside at the nodes (red boxes) of the 3-D grid (white lines). There they can interact with other agents and move to neighboring nodes. Agents are assessed at regular, specified time intervals as they move and interact upon the grid. Virtual cells were allowed to leave the Waldeyer ring via draining lymphatics and return via the peripheral blood and HEVs (Figure 2A and 2B; Video S1) as in normal mucosal lymphoid tissue [19]. A brief summary list of the agents employed in our simulation, and their properties and interactions, is given in Table 1. In this report we refer to actual B cells as, for example, “B cells”, “latently infected B cells”, or “lytically infected B cells”, and their virtual representations as “virtual B cells”, “B_Lat_s”, or “B_Lyt_s”. Similarly, we refer to actual virus as virions and their virtual counterparts as virtual virus or Vir. A full description of the simulation, including a complete list of agents, rules, the default parameters that produce the output described below, and a preliminary survey of the extended parameter space is presented in M. Shapiro, K. Duca, E. Delgado-Eckert, V. Hadinoto, A. Jarrah, et al. (2007) A virtual look at Epstein-Barr virus infection: simulation mechanism (unpublished data). Here, we will first present a description of how the virtual environment was visualized and then focus on a comparison of simulation output with the known biological behavior of the virus.

**Table 1:**
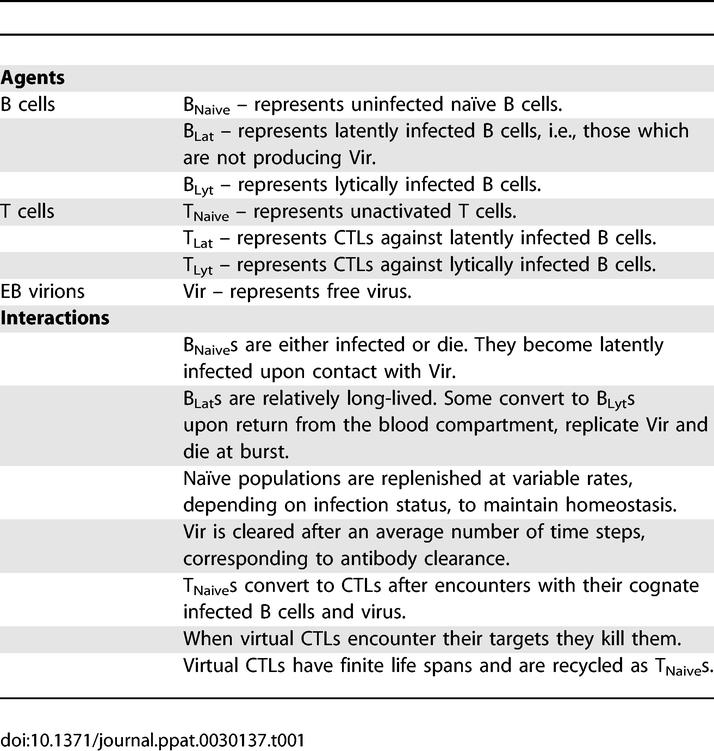
A Summary of the Agents and Their Governing Rules Used in This Simulation

### The Virtual Environment

Simulation runs were accessed through an information-rich virtual environment (IRVE) (Figures 2 and 3; Videos S1 and S2), which was invoked through a Web interface. This provided a visually familiar, straightforward context for immediate comprehension of the spatial behavior of the system [20]. It also allowed specification of parameters, run management, and ready access to data output and analysis.

Figure 3 demonstrates how the time course of infection may be visualized. Usually the simulation was initialized by a uniform distribution of Vir over the entire surface of the Waldeyer ring, thereby seeding infection uniformly. However, in the simulation shown in Figure 3A, virtual EBV was uniformly deposited only on the lingual tonsil. Figure 3B–3D shows the gradual spread of virtual infection (intensity of red color indicating the level of free Vir) to the adjacent tonsils. It can be seen in this case that the infection spreads uniformly to all the tonsils at once, implying that it was spreading via B_Lat_s returning from the blood compartment and reactivating to become B_Lyt_s, rather than spreading within the ring. Examples of infectious spread between and within the tonsils can also be seen in Video S2.

### Simulation Output and Validation

#### Time course of infection.

**Figure 4:**
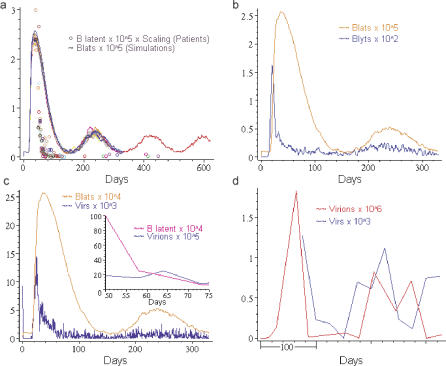
Output of the Simulation Employing the Default Parameter Set Matched to Biological Data (A) Simulation output of the total number of latently infected virtual B cells (B_Lat_) over time in a series of superimposed simulation runs (colored lines) versus the number of actual latently infected memory B cells in the peripheral blood of 15 acutely infected individuals over time (colored circles). The first patient time point is assumed to be at 50 d +/− 10 d (see text for explanation). The simulation runs all use the default parameter set. Stochasticity (i.e., random variations) in the runs are a consequence of the fact that every interaction and motion of agents is governed by a probability and a variability function. So, for example, the initial infectious dose will vary from node to node in the epithelium and will have a certain average. This average we refer to as the initial infectious dose. For a detailed discussion of all the rules governing interactions and motion, see M. Shapiro, K. Duca, E. Delgado-Eckert, V. Hadinoto, A. Jarrah, et al. (2007) A virtual look at Epstein-Barr virus infection: simulation mechanism (unpublished data). (B) Simulation output of the total number of latently infected virtual B cells (B_Lat_) and virtual B cells replicating virtual virus (B_Lyt_) over time for a single simulation run. (C) Simulation output of the level of latently infected virtual B cells (B_Lat_) and free virtual virus (Vir) over time for a single simulation run. The insert shows a time course for a typical acutely infected patient for the first 25 d after entering the clinic showing the level of latently infected memory B cells in the peripheral blood and free virions shed into the saliva. (D) The level of virtual free virus (Vir) over 1 y in the simulation (blue line). The level was assessed every 25 d, once the simulation had entered into the persistent phase defined as a stable level of latently infected virtual B cells (B_Lat_) For comparison, the level of shed virus (Virions) in the saliva of a persistently infected individual, sampled on average approximately every 30 d, is shown (red line). The *x*-axis denotes the number of days over which sampling was performed and does not relate to the time from initial infection N.B. Note this figure only allows analysis of overall dynamics, not absolute numbers, since all comparisons between simulation and in vivo data are relative.

There were a number of properties of the initial infection that were in agreement when natural EBV infection was compared to the simulation. Typical output from the simulation, employing the default parameter set, is shown in Figure 4 along with relevant biological data for comparison. Figure 4A shows the time course for the number of B_Lat_s taken from multiple simulation runs. Superimposed upon this are data from 15 individuals acutely infected with EBV (AIM patients). The simulation showed several features with striking similarity to natural infection (for a summary see Table 2). First, there was a peak of infected cells that approached up to 50% of the virtual B cells. This would seem unlikely; however, we have previously documented that at the first clinic visit by AIM patients the number of infected memory B cells in the periphery can indeed approach 50% [17]. Second, the peak of virtual infected B cells (B_Lat_s) occurred at 33–38 d. This was in good agreement with the published incubation period for AIM of 35–50 d [21]. Third, there was a dramatic decline in the levels of virtual infected cells that resolved into low-level persistent infection that continued for the longest time period we have examined (∼3 y). We define persistence here as the normal course of EBV infection, i.e., an acute infection that clearly resolves into a long-term stable presence of much lower levels of viral genomes both in latently infected cells and as free virus. The overall decline in virtual infected cells (and Vir production, see below) appeared to proceed more and more slowly rather than come to a steady state, at least for the time periods we have simulated. This behavior was compatible with that seen in recent kinetic studies of acute EBV infection [17].

**Table 2:**
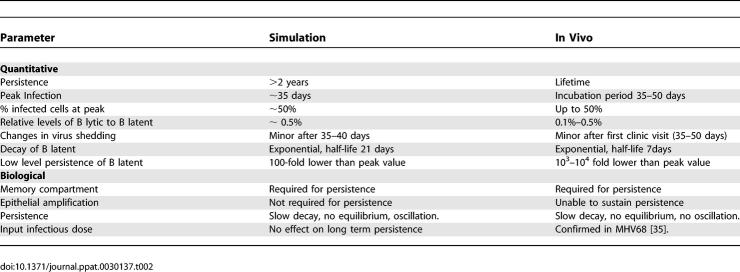
Validation of Simulation Output

The simulation was less precise in the absolute quantitation of recovery. During the acute phase of AIM, the levels of infected cells drop rapidly, with a half-life of approximately 7 d (V. Hadinoto, M. Shapiro, T. Greenough, J. Sullivan, K. Luzuriaga, D. Thorley-Lawson (2007) On the dynamics of acute EBV infection and the pathogenesis of infectious mononucleosis (unpublished data)), to a level that is 10^3^-fold lower in 6 mo [17] and ultimately 10^4^- to 10^5^-fold lower during persistent infection [13]. By comparison, virtual infection resolved significantly more slowly in the simulation, with a half-life of ∼21 d, and less well, with only ∼ a 20-fold drop by 6 mo. This was followed by a marked oscillation with a frequency of around 6 mo to 1 y and a drop of no more than 100-fold at the 1 y nadir. It is conceivable that oscillations occur during natural infection with similar frequencies to those seen in the simulation, but they would have to occur with much smaller amplitudes since they have not been seen in our studies to date (V. Hadinoto and D. Thorley-Lawson, unpublished data).

An important caveat to these comparisons is that the simulation presents the dynamics of infection in the Waldeyer ring and the peripheral circulation, whereas the dynamics of EBV infection in vivo has only been studied in the peripheral blood and saliva. However, this is probably not a major concern since we have shown previously that, at least during persistent infection, the levels of infected B cells in the peripheral blood and Waldeyer ring are comparable [13]. Thus, in terms of general quantitative and qualitative features, this is the first simulation to our knowledge to reproduce both the acute and persistent phases of latent EBV infection.

#### Viral replication and production.

Another feature where the simulation represented the known features of EBV infection is Vir replication and production, which continued at a low level throughout the persistent phase (Figure 4B and 4C). Furthermore, the fraction of virtual B cells replicating Vir (∼0.05%–0.1% in both acute and persistent phase) was remarkably similar to what we have estimated for the tonsils of persistently infected individuals (∼0.05%–0.1% complete the replicative cycle) [16]. One unusual feature of AIM was that, while the levels of latently infected B cells were decreasing exponentially for the first 5–7 wk after the patient had arrived in the clinic, the level of virus production by the lymphoepithelium was already quite stable, declining very little over the same time period ([22,23] and V. Hadinoto, M. Shapiro, T. Greenough, J. Sullivan, K. Luzuriaga, et al. (2007) On the dynamics of acute EBV infection and the pathogenesis of infectious mononucleosis (unpublished data)). This feature was also observed in the simulation if we assume that individuals enter the clinic at or soon after peak infection of B cells. Figure 4C compares the overall dynamics of B_Lat_ and virtual virus (Vir) production during a simulated infection. By day 50, Vir production had already declined close to persistent levels, whereas the number of B_Lat_ was still falling exponentially and continued to do so for approximately another 50 d. The insert in Figure 4C shows comparative representative data from a single AIM patient. In this case, again, virion shedding remained relatively stable for at least 60 d (Figure 4C and not shown) after the first clinic visit, whereas the levels of latently infected memory B cells fell rapidly. As before, the levels of latently infected memory B cells fell much more rapidly in actual infection compared to the simulation.

The simulation was also accurate in modeling certain detailed aspects of virus production. Thus, during a real persistent infection, virus production, as judged by shedding into saliva, was highly variable (V. Hadinoto and D. Thorley-Lawson, unpublished data), with ≥3 logs difference over the course of a year (Figure 4D, range from 7.3 × 10^2^ to 1.8 × 10^7^ virions/ml of saliva), whereas we have shown previously that the level of latently infected B cells in the blood is relatively stable for years [18]. When we looked at similarly spaced time points from a simulation run, we found the same degree of variation in Vir production (Figure 4D, range 1 × 10^3^ to 1.3 × 10^3^) while the level of B_Lat_s over a similar period was again quite stable (e.g., compare B_Lat_ and Vir production in Figure 4C).

There is also a caveat to these comparisons. The simulation reports the total number of Vir present, whereas in a real infection, we can only measure viral shedding in the saliva. We make the assumption that these two events are at least proportionally related to each other. Given this caveat, we can conclude that the simulation also reproduces the overall dynamics of viral replication during infection. We can extend this claim to conclude that these studies provide the first evidence to our knowledge that the simulation has predictive power, because the observations of stochasticity in virus production and of the discrepancy in the time of stabilization for the levels of virus shedding and B latent were seen in the simulation before being tested and observed experimentally.

#### The peripheral memory compartment is required for long-term persistence.

**Figure 5:**
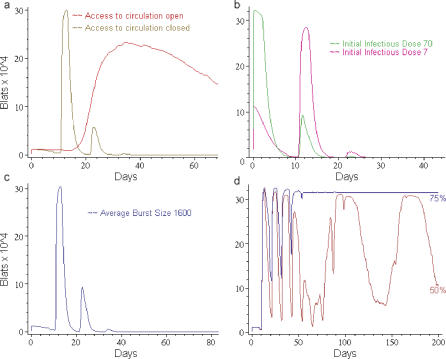
Access to the Peripheral Circulation Is Required for Viral Persistence in the Simulation (A) The virtual virus does not persist when latently infected virtual B cells (B_Lat_) are denied access to the peripheral circulation. The graph shows the level of B_Lat_s over time for a simulation run with the default parameter set allowing (red line) or denying (brown line) access of the B_Lat_s to the peripheral circulation Note that only the first 60 d of virtual infection are shown. A characteristic series of spikes in B_Lat_s is observed that was lost after approximately 30–40 d. These spikes arise because normally B_Lat_s spend time in the circulation, and the time they spend there does not advance them towards the lytic state. By denying them access to the blood, both the mean time to burst and the standard deviation of this statistic decrease. This causes the virtual viral bursts and infection of new virtual B cells to arrive synchronously as narrow spikes, one spike per generation of infected virtual cells. (B) Increasing the initial infectious dose does not allow persistence in the absence of a peripheral blood compartment. The simulation was run without a peripheral blood compartment as described in (A), except the initial infectious dose was increased in increments as shown. The initial infectious dose is the average number of Vir deposited at each mesh point (node) on the surface of the virtual lymphoepithelium. To obtain the total infectious dose, this value is multiplied by the number of surface nodes (16,205). The figure shows runs with infectious dose of 7/mesh point (pink line) and 70/mesh point (green line). The default value for initial infectious dose is 0.7/mesh point. (C) Increasing the Vir burst size does not allow persistence in the absence of a peripheral blood compartment. The simulation was run without a peripheral blood compartment as described in (A), except the number of Virs released when a B_Lyt_ bursts was doubled (default average burst size = 800 Virs). (D) Decreasing the efficiency of the immune response does not allow persistence in the absence of a peripheral blood compartment. The simulation was run without a peripheral blood compartment as described in (A), except the efficiency with which CTLs are activated and kill their targets was reduced incrementally. With this approach it was possible to find a level of virtual immunosuppression (50% brown line) that produced an infection that lasted for the length of the simulation (880 virtual days). However, this looks nothing like EBV persistence in a real infection. Rather, the virtual infection involves wild oscillations in the number of latently infected virtual B cells (B_Lat_) and it appears that the average level is trending upwards to overwhelm the B cell compartment, a condition we refer to as virtual death. Small incremental increases above 50% immunosuppression (e.g., 75% blue line) resulted in the B cell compartment being rapidly overwhelmed with latently infected virtual B cells (B_Lat_). Small incremental decreases below 50% immunosuppression resulted in clearance of Vir similar to that seen in Figure 5A.

One advantage of a simulation strategy is that it allows us to track the consequences of altering conditions and parameters that would not necessarily be feasible in a human. A central, but unproven, tenet of the EBV model is that the pool of immunologically invisible, latently infected memory B cells in the peripheral circulation is the site of and essential for lifetime persistence. To address this question *in silico*, we ran the simulation while maintaining the default parameter set, but prevented access of infected B_Lat_s to the peripheral circulation. In this case, we observed a characteristic series of spikes in B_Lat_s that was lost after approximately 30–40 d. (Figure 5A). The explanation for these spikes is that in a normal run, B_Lat_s spend time in the circulation, and the time they spend there does not advance them towards the lytic state. By denying them access to the blood, both the mean time to burst and the standard deviation of this statistic decrease. This causes the virtual viral bursts and infection of new virtual B cells to arrive as narrow spikes, one spike per generation of infected virtual cells.

Since the maintenance of persistent infection appeared dependent on the availability of a peripheral compartment, we wondered how robust this dependence was. To address this we attempted to compensate for lack of access to the circulation by raising either the initial infectious dose of Vir or the burst size or by weakening the immune system (Figure 5B–5D). Raising the initial infectious dose (Figure 5B) or burst size (Figure 5C) had little impact on the overall dynamics, with both still leading to clearance, albeit increased burst size did delay this by about 30 d. It is conceivable that by increasing burst size even further a virtual infection could be generated where virtual virus was still present at the end of the simulation run; however, it would require highly unphysiological levels of bursting and generate an output consisting of wild oscillations with a periodicity of about 10 d, nothing like real EBV persistence. One counterintuitive observation was that increasing the initial infectious dose actually led to a reduction in the peak of acute infection. The explanation for this is that the higher initial levels of Vir stimulate a more aggressive immune response. A similar phenomenon is seen in the presence of a peripheral compartment (see below).

Reduction of CTL function by 75% (Figure 5D, blue line) or more resulted in an acute accumulation of B_Lat_, which rapidly and irreversibly overwhelms the B cell compartment. By carefully titrating back the virtual immunosuppression to 50% (Figure 5D, brown line), it was possible to attain an infection that had not cleared nor overwhelmed the B cell compartment by the end of the run (880 d). In this case, we see large oscillations of the B_Lat_ population with a very slow trend upward, suggesting that the B cell compartment will eventually be overwhelmed. It was interesting to note that the periodicity of these large swings (75–100 d) is similar to that seen for chronic fatigue syndrome [24], which had originally been linked to EBV, but for which causation remains unclear. In this syndrome, patients develop symptoms of an acute virus infection every few months. Our simulation output raises the possibility that chronic fatigue syndrome could be caused by a virus or viruses like EBV that are denied full access to their site of persistence, where they are no longer under immunosurveillance, in the presence of mild immunosuppression such as stress, which is a known co-factor in chronic fatigue syndrome.

In summary, for the range of parameters we have tested, it was not possible to obtain a virtual persistent infection characteristic of EBV in the absence of a peripheral B cell pool. This suggests that access of B_Lats_ to the peripheral pool, the equivalent to the circulating memory B latent, is required for persistent infection and is consistent with the clinical observations that the B cell compartment is essential for persistence [25,26].

#### Effect of initial infectious dose on long-term persistence.

**Figure 6:**
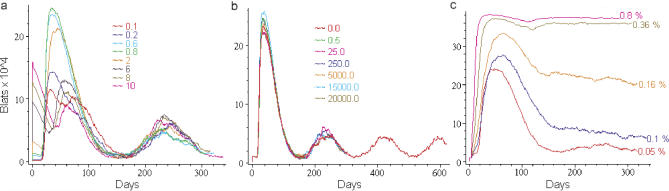
Comparing Simulation Output, Employing the Default Parameter Set, to What Is Known or Expected from a Real Infection (A) Varying the initial infectious dose of Vir has no effect on persistent levels of latently infected virtual B cells (B_Lat_). The initial infectious dose is the average number of Vir deposited at each mesh point (node) on the surface of the virtual lymphoepithelium. The simulation was run as described in Figure 4A, except the initial infectious dose of Vir was varied from 0.1 to 10 as shown (the default level is 0.7). Since there are 16,205 surface mesh points, this translates into a total infectious dose ranging from 1.6 × 10^3^ to 1.6 × 10^5^. (B) Epithelial cell amplification has no effect on persistent levels of latently infected virtual B cells (B_Lat_). Epithelial cell amplification of Vir was simulated by adding an additional amount of Vir at each time step. The virtual amplification achieved varied from none to 2 × 10^4^. (C) Persistence is highly sensitive to the rate of Vir reactivation. The simulation was run as described in Figure 4A, except the percentage of latently infected virtual B cells (B_Lat_) that initiate Vir replication upon return to the Waldeyer ring was varied as shown (the default percentage is 0.05% based on data from [16]).

We wanted to test how robust persistence was in the presence of the blood compartment, i.e., had we fortuitously picked just the right conditions for persistence. We found that it was robust over a wide range of parameters. Two examples are given in Figure 6A and 6B. First, we tested the outcome of varying the initial infectious dose of Vir from 10^3^–10^6^ (default value ≈ 5 × 10^4^). For comparative purposes we note that we detect ∼10^4^–10^7^ viral genomes in thorough mouth rinses from healthy carriers of EBV (V. Hadinoto and D. Thorley-Lawson, unpublished data). Thus, the range we have tested would be consistent with 10% of actual virions in the saliva being infectious, which may be an overestimate. What we observed (Figure 6A) was that the level of input Vir affected the level of acute infection but had no impact on the level of persistent infection. An interesting feature was that as the initial infectious dose increased, the severity of acute infection tended to decrease as measured by the level of B_Lat_. This happens because the higher dose provokes a more aggressive immune response that controls the infection process more efficiently. This observation raises the interesting possibility that a low dose of infectious virus may actually be more likely to contribute to development of AIM than a high dose. There are many possible biological explanations for how this variation in infectious dose could actually arise. For example, the permeability of the epithelial layer to the virus may be greater in children than adults, allowing a much smaller fraction of the infectious dose access to the lymphoid compartment in adults.

Although it is generally believed that EBV persists in the mature B lymphoid compartment, there is a growing consensus that it also infects epithelial cells in the nasopharyngeal region [27–29]. The role of this type of infection is unclear but likely to involve amplification of the virus since EBV infection of epithelial cells seems to give rise to high level replication rather than long-term latency [30]. A contribution of the epithelial compartment to Vir production was not included in the initial simulation; however, a revised simulation with epithelial amplification of Vir was constructed by introducing additional Vir at the epithelial surface at each time step (* in Figure 1). Effectively, this was also a way to increase input Vir. Again, it had virtually no impact on the long-term levels of B_Lat_ over a range of 4 logs in amplification (Figure 6B).

In summary, these results predict that the level of B cell infection during persistence will be independent of the starting dose of input virus and that epithelial amplification is not required to establish or maintain persistent infection.

#### The fraction of B_Lats_ reactivating upon return to the Waldeyer ring represents a critical switch point.

One critical feature to which the establishment of virtual persistence was very sensitive was the rate at which B_Lats_ become B_Lyts_ and begin producing new progeny Vir upon return from the blood to the Waldeyer ring. As this rate was increased, there was a sharp switch point where the virtual infection no longer declined into persistence but overwhelmed the virtual B cell compartment with infected virtual B cells, leading to what we term virtual death within 2 wk (Figure 6C). This type of catastrophic acute infection leading to rapid death is typical of the disease course of the fatal acute EBV infection X-linked lymphoproliferative disease [12] and provides an example of how a relatively small change in a biological parameter at a critical time in the infection can dramatically change the outcome. It was also striking that the maximal fraction of returning B_Lats_ that could reactivate to B_Lyts_ and still allow the establishment of persistence (∼0.1%–0.2%) was very close to, but slightly higher than, the fraction we have actually measured in the tonsils of healthy carriers (∼0.05%–0.1% complete the replicative cycle) [16]. This raises the possibility that host and virus have co-evolved to allow the maximal amount of viral reactivation possible, presumably to maximize the chance of virus spread to new hosts, without putting the host at risk for fatal acute infection that would result in the death of both host and virus.

## Discussion

In this paper we present a comprehensive model of EBV infection that effectively simulates the overall dynamics of acute and persistent infection. The fact that this simulation can be tuned to produce the course of EBV infection suggests that it models the basic processes of this disease. To achieve this, we have created a readily accessible, virtual environment that appears to capture most of the salient features of the lymphoid system necessary to model EBV infection. Achieving infection dynamics that reflect an acute infection followed by recovery to long-term low-level persistent infection seems to require access of the virus to a blood compartment where it is shielded from immunosurveillance. Because we cannot perform a comprehensive parameter search (due to the very large parameter space involved), we cannot unequivocally state that the blood compartment is essential. What is clear though, is that persistence is a very robust feature in the presence of a blood compartment, and that we could not achieve an infection process that even remotely resembles typical persistent EBV infection in its absence.

The areas in which the simulation most closely follows known biology are summarized in Table 2 and include the peak time of infection, 33–38 d, compared to the incubation time for AIM of 35–50 d [21]. This predicts that patients become sick and enter the clinic at or shortly after peak infection in the peripheral blood, a prediction confirmed by our patient studies, where the numbers of infected B cells in the periphery always decline after the first visit [17].

An important feature of a simulation is its predictive power. Our analysis predicted that access to the peripheral memory compartment is essential for long-term persistence. This is consistent with recent studies on patients with hyper-IgM syndrome [31]. Although these individuals lack classical memory cells, they can be infected by EBV; however, they cannot sustain persistent infection and the virus becomes undetectable. Unfortunately, those studies did not include a sufficiently detailed time course to see if time to virus loss coincided with the simulation prediction of 1–2 mo.

Another area where the simulation demonstrated its predictive power was in the dynamics of viral replication. In the simulation it was unexpectedly observed that the level of Vir production plateaued long before B_Lats_, predicting that the levels of virus shedding, unlike latently infected cells, will have leveled off by the time AIM patients arrive in the clinic. This prediction, which contradicted the common wisdom that virus shedding should be high and decline rapidly in AIM patients, was subsequently confirmed experimentally (V. Hadinoto, M. Shapiro, T. Greenough, J. Sullivan, K. Luzuriaga, D. Thorley-Lawson (2007) On the dynamics of acute EBV infection and the pathogenesis of infectious mononucleosis (unpublished data) and see also [22,23]). The simulation also quite accurately reproduces the relatively large variation in virus production over time, compared to the stability of B latent. This difference is likely a consequence of stochasticity (random variation) having a relatively larger impact on virus production. This is because the number of B cells replicating the virus at any given time is very small, both in reality and the simulation, compared to the number of infected B cells, but the number of virions they release when they do burst is very large. This difference may reflect on the biological requirements for persistence of the virus since a transient loss in virus production due to stochasticity can readily be overcome through recruitment from the pool of B latents. However, a transient loss of B latents would mean clearance of the virus. Hence, close regulation of B latent but not virion levels is necessary to ensure persistent infection.

Although there is now a growing consensus that EBV infects normal epithelial cells in vivo [27–29], the biological significance of this infection remains unclear. The available evidence suggests that epithelial cell infection may not be required for long-term persistence [25,26], and this is also seen in the simulation. The alternate proposal is that epithelial infection might play an important role in amplifying the virus, during ingress and/or egress, as an intermediary step between B cells and saliva. This is based on the observation that the virus can replicate aggressively in primary epithelial cells in vivo [30]. In the simulation, epithelial amplification had no significant effect on the ability of Vir to establish persistence. This predicts that epithelial amplification does not play a critical role in entry of the virus, but leaves open the possibility that it may be important for increasing the infectious dose present in saliva for more efficient infection of new hosts.

The simulation is less accurate in the precise quantitation of the dynamics. Virtual acute infection resolves significantly more slowly and persistence is at a higher level than in a real infection. In addition, virtual persistent infection demonstrates clear evidence of oscillations in the levels of infected cells that have not been detected in a real infection. The most likely explanation for these discrepancies is that we have not yet implemented T cell memory. Thus, as the levels of virtual infected cells drops, the immune response weakens, allowing Vir to rebound while a new supply of virtual CTLs is generated. Immunological memory would allow a more sustained T cell response that would produce a more rapid decline of infected cells, lower levels of sustained persistence, and tend to flatten out oscillatory behavior, thus making the simulation more quantitatively accurate. This is one of the features that will be incorporated into the next version of our simulation. It remains to be determined what additional features need to be implemented to sharpen the model and also whether and to what extent the level of representation we have chosen is necessary for faithful representation of EBV infection. Our simulation of the Waldeyer ring and the peripheral circulation was constructed with the intent of modeling EBV infection. Conversely, our analysis can be thought of as the use of EBV to validate the accuracy of our Waldeyer ring/peripheral circulation simulation and to evaluate whether it can be applied to other pathogens. Of particular interest is the mouse gamma herpesvirus MHV68 [32,33]. The applicability of MHV68 as a model for EBV is controversial. Although it also persists in memory B cells [34], it appears to lack the sophisticated and complicated latency states that EBV uses to access this compartment. However, one of the simplifications in our simulation is that the details of these different latency states and their transitions are all encompassed within a single concept, the B_Lat_. We have also assumed a time line whereby a newly infected B_Lat_ becomes activated and CTL sensitive, migrates to the follicle, and exits into the circulation, where it is no longer seen by our virtual CTLs. In essence, we have generalized the process by which the virus proceeds from free virion to the site of persistence in such a way that it may be applicable to both EBV and MHV68. Thus, we might expect that the overall dynamics of infection may be similar even though detailed biology may vary. As a first step to test if this concept had value, we performed an analysis based on studies with MHV68 where it was observed that the levels of infected B cells at persistence were unaffected by the absolute amount of input virus at the time of infection [35]. When this parameter was varied in the simulation, we saw the same outcome. This preliminary attempt raises the possibility that the mouse virus may be useful for examining quantitative aspects of EBV infection dynamics.

The last area we wished to investigate was whether we could identify biologically meaningful “switch” points, i.e., places in time and space where relatively small changes in critical parameters dramatically affect outcome, for example, switching from persistence to clearance to death. We have observed one such switch point—reactivation of B_Lats_ upon return to the Waldeyer ring—that rapidly switches the infection process from persistence to death. How this might relate to fatal EBV infection, X-linked lymphoproliferative disease, is uncertain. However, viral production is a function both of how many B cells initiate reactivation and how efficiently they complete the process. We believe that most such cells are killed by the immune response before they release virus [16], so defects in the immune response could allow more cells to complete the viral replication process and give the same fatal outcome.

The ability to find such conditions for switch points could be very useful in the long term for identifying places in the infection process where the virus might be optimally vulnerable to drug intervention. The easiest place to target EBV is during viral replication; however, it is currently unclear whether viral replication and infection are required for persistence. It may be that simply turning off viral replication after persistence is established fails to eliminate the virus because the absence of new cells entering the pool through infection is counterbalanced by the failure of infected cells to disappear through reactivation of the virus. If, however, a drug allowed abortive reactivation, then cells would die without producing infectious virus and new infection would be prevented. This models the situation that would arise with a highly effective drug or viral mutant that blocked a critical stage in virion production (e.g., viral DNA synthesis or packaging), so that reactivation caused cell death without release of infectious virus. A similar effect could be expected with a drug or vaccine that effectively blocked all new infection. This is another case in which studies with the mouse virus, where non-replicative mutants can be produced and tested, may be informative as to whether and to what extent infection is required to sustain the pool of latently infected B cells and persistence. The simulation could then be used to predict how effective an anti-viral that blocked replication, or a vaccine that induced neutralizing antibodies, would need to be at reducing new infection in order to cause EBV to be lost from the memory pool (for a more detailed discussion of this issue see M. Shapiro, K. Duca, E. Delgado-Eckert, V. Hadinoto, A. Jarrah, et al. (2007) A virtual look at Epstein-Barr virus infection: simulation mechanism (unpublished data)).

Most modeling of virus infection to date has tended to focus on HIV and use differential equations [2–7]. One such study involved EBV infection [36], but to our knowledge none outside of our group has addressed the issue studied here of acute EBV infection and how it resolves into lifetime persistence. In preliminary studies of our own, modeling EBV infection with differential equations that incorporate features common to the HIV models, with parameters physiologically reasonable for EBV did not produce credible dynamics of infection (K. Duca, unpublished observations). Although we do not exclude the possibility that such models may be useful for simulating EBV, we took an agent-based approach because it is intuitively more attractive to biologists. Such models are increasingly being recognized as an effective alternative way to simulate biological processes [37–39] and have several advantages. The main advantage is that the “agent” paradigm complies by definition with the discrete and finite character of biological structures and entities such as organs, cells, and pathogens. This makes it more accurate, from the point of view of scientific modeling. It is also less abstract since the simulated objects, processes, and interactions usually have a straightforward biological interpretation and the spatial structure of the anatomy can be modeled meticulously. The stochasticity inherent to chemical and biological processes can be incorporated in a natural way. Lastly, it is generally much easier to incorporate qualitative or semi-quantiative information into rule sets for discrete models than it is for such data to be converted to accurate rate equations. The major drawback to agent-based models is that there is currently no mathematical theory that allows for rigorous analysis of their dynamics. Currently, one simply runs such simulations many times and performs statistical analyses to assess their likely behaviors. Developing such a mathematical theory remains an important goal in the field.

In summary, we have described a new computer simulation of EBV infection that captures many of the salient features of acute and persistent infection. We believe that this approach, combined with mouse modeling (MHV68) and EBV studies in patients and healthy carriers, will allow us to develop a more profound understanding of the mechanism of viral persistence and how such infections might be treated and ultimately cleared.

## Methods

### Biological data.

Details of the AIM patient populations tested have been published previously [17]. Adolescents (ages 17–24) presenting to the clinic at the University of Massachusetts at Amherst Student Health Service (Amherst, Massachusetts, United States) with clinical symptoms consistent with acute infectious mononucleosis were recruited for this study. Following informed consent, blood and saliva samples were collected at presentation and periodically thereafter. Diagnosis at the time of presentation to the clinic required a positive monospot test and the presence of atypical lymphocytes [21]. Confirmation of primary Epstein–Barr infection required the detection of IgM antibodies to the EBV viral capsid antigen in patient sera [40]. These studies were approved by the Human Studies Committee at the University of Massachusetts Medical School (Worcester, Massachusetts, United States) and by the Tufts New England Medical Center and Tufts University Health Sciences Institutional Review Board. All blood samples were diluted 1:1 in 1x PBS.

The technique for estimating the absolute number of latently infected B cells in the peripheral blood of patients and healthy carriers of the virus is a real-time PCR–based variation of our previously published technique [17], the details of which will be published elsewhere (V. Hadinoto, M. Shapiro, T. Greenough, J. Sullivan, K. Luzuriaga, et al. (2007) On the dynamics of acute EBV infection and the origins of infectious mononucleosis (unpublished data)). To measure the absolute levels of virus shedding in saliva, individuals were asked to rinse and gargle for a few minutes with 5 ml of water and the resultant wash processed for EBV-specific DNA PCR using the same real-time–based PCR technique. We have performed extensive studies to standardize this procedure that will be detailed elsewhere (V. Hadinoto, M. Shapiro, T. Greenough, J. Sullivan, K. Luzuriaga, et al. (2007) On the dynamics of acute EBV infection and the origins of infectious mononucleosis (unpublished data)).

### The simulation.

In the simulation, B cells are either uninfected (B_Naïve_), latently infected (B_Lat_), or replicating virtual virus (B_Lyt_); we do not distinguish blast and memory B cells. In the biological model, newly infected B cells in the lymphoepithelium of the Waldeyer ring pass through different latency states, which are vulnerable to attack by cytotoxic T cells (CTL latent). Subsequently, they become memory B cells that enter the peripheral circulation and become invisible to the immune response by turning off viral protein expression. In the simulation, all these latency states are captured in the form of a single entity, the B_Lat_. In addition, the blood circulation and lymphatic system are both represented as abstract entities that only allow for transport of B_Naïves_ and B_Lats_ around the body. Virtual T cells are restricted to the Waldeyer ring. This simplification is based on the assumption that, in the biological model, EBV-infected cells entering the peripheral circulation are normal and invisible to CTLs, because the virus is inactive, and therefore the peripheral circulation simply acts as an independent pool of and a conduit for B latent. Operationally, therefore, B_Lats_ escape T_Lats_ in the simulation simply by entering the peripheral circulation. Consequently, unlike the biological model, B_Lats_ are vulnerable to T_Lats_ whenever they reenter the lymph node.

Each agent (e.g., Vir or a B_Naïve_) has a defined life span, instructions for movement, and functions that depend on which other agents they encounter (for example, if a Vir encounters a B_Naïve_, it infects it with some defined probability). The agents, rules, and parameters used are based on known biology wherever possible with simplifications (see above) where deemed appropriate. A brief description and discussion of the agents and their rules is given in Table 1. A detailed listing is provided in M. Shapiro, K. Duca, E. Delgado-Eckert, V. Hadinoto, A. Jarrah, et al. (2007) A virtual look at Epstein-Barr virus infection: simulation mechanism (unpublished data). At each time point (6 min of real time), every agent is evaluated and appropriate actions are initiated.

### The IRVE.

The simulation is invoked through a Web interface (IRVE; see movies linked to Figure 2, and [20]) that allows a straightforward visual, familiar, and scalable context for access to parameter specification, run management, data output, and analysis. This has the additional advantage that it readily allows comprehension of the spatial behavior of the system (e.g., “how does the infection spread?”). The simulation may also be invoked from the command line. Through the Web, users can process simulation data for output and analysis by a number of common applications such as Microsoft's Excel, University of Maryland's TimeSearcher [41], and MatLab.

We have developed display components that encapsulate multiple-view capabilities and improved multi-scale interface mappings. The IRVE is realized in the international standard VRML97 language. The simulation can be rerun and reanalyzed using a normal VCR-type control tool, which allows the operator, for example, to fast forward, pause, rewind, or drag to a different time point, and to play back runs or analyze simulation output dynamically. In the IRVE, any spatial object (including the global system) can be annotated with absolute population numbers (as a time plot and/or numeric table) or proportional population numbers (as a bar graph) for any or all of the agents. Spatial objects themselves can be animated by heat-map color scales. The intensity of the color associated with each agent is a measure of the absolute level of the agent; so, for example, as the level of free Vir increases, so will the level of intensity of the associated color (in this case red) both within the single units and in the entire organ.

In our simulation we manage multiple views of the dynamic population values through a higher order annotation called a PopView (population view). A PopView is an interactive annotation that provides three complementary representations of the agent population. The representations can be switched through in series by simple selection. The default view is a color-coded bar graph where users can get a quick, qualitative understanding of the agent populations in a certain location at that time step. The second is a field-value pair text panel, which provides numeric readouts of population levels at that time step. The third is a line graph where the population values for that region are plotted over time.

Because of the large amount of time points and the large number of grid locations, the IRVE manages an integrated information environment across two orders of magnitude: “Macro” and “Micro” scales. Through the standard VRML application the user has a number of options including free-navigational modes such as: fly, pan, turn, and examine. This allows users to explore the system, zooming in and out of anatomical structures as desired. In addition, the resulting visualization space is navigable by predefined viewpoints, which can be visited sequentially or randomly through menu activation. This guarantees that all content can be accessible and users can recover from any disorientation. The Visualizer manages Macro and Micro scale result visualizations using proximity-based filtering and scripting of scene logic. As users approach a given anatomical structure, the micro-scale meshes and results are loaded and synchronized to the time on the users' VCR controller.

## Supporting Information

Video S1 Click here for additional data file. The Fine Structure of the Simulation Grid Visualized through the IRVE
In this video, a single tonsil, the right tubal, is initially viewed as a collection of 144 of the hex units that are the basic component of the simulation. The video pans and then zooms in to reveal the fine structure of each unit and the grid that composes it. The tissue structures represented are shown in detail, including the single HEV entry point from the peripheral circulation and the exit point to the lymphatics. The video then zooms into the tonsil, revealing the network along which the agents move (represented as white lines) and the nodes where the agents reside and interact (red boxes).
(68.4 MB AVI)

Video S2 Click here for additional data file. An Overview of the Simulation Visualized through the IRVE
This video demonstrates the flexibility available through the motion tools to enter and move around the simulation and through the graphics tools to access data. It begins with a whole body view with the general control panel for running the simulation shown at the bottom. The global levels of different agents are represented as color-coded bar graphs. Red represents Vir, green uninfected naïve B cells, yellow B_Lat_, white B_Lyt_ dark blue naïve T cells, purple CTL_Lat_, and light blue CTL_Lyt_. The controls are similar to a typical VCR control. It allows the operator to fast forward, pause, rewind, or drag the blue cursor to a different time point and the capability to play back runs or analyze simulation output dynamically. The pointer then reveals how with a simple click the global statistics can be switched from a bar graph to numeric values to a time-dependent line graph. The pointer is then used to show how the data graphs can be easily rotated, magnified, or dragged to a different location for convenient viewing. The pointer is then used to drag the blue cursor on the control panel and it can be seen that an equivalent cursor moves across the line graph, allowing precise selection of the time step to be viewed.
The view then moves to the next layer, the Waldeyer ring in the skull, whilst demonstrating that graphic data can be obtained at any level of the simulation. The tonsils, adenoids, and connecting tissue are seen along with the access for the peripheral circulation (red and blue vessels) and lymphatics (white vessels). The image demonstrates the versatility of the simulation, allowing motion into and through the Waldeyer ring whilst at any time selecting data presentations for each compartment or for the global values. The color of the ring indicates the agent being monitored and its intensity the amount. Thus, when the selection switches to red, we observe Vir levels just after peak infection. The blue cursor is dragged back in time to the beginning and the increase and subsequent decrease in Vir are visualized by the change in color intensity. Meanwhile, the qualitative information provided by the color intensity can be precisely quantitated at any time by selecting the appropriate data box. The selected color then changes to white, allowing the level of B_Lyt_ to be monitored as the infection spreads and then run back simply by dragging the cursor. The selected color then changes to purple allowing the spread of CTL_Lyt_ to be monitored as the cursor is dragged.
The video then zooms into a single tonsil, the right palatine, while continuing to demonstrate that local and global data can always be accessed. The initial shot shows the saturation of the tonsil with Vir. The cursor is then dragged back and forth to the beginning, demonstrating the changes in level of Vir over time. The video then pans sideways to reveal that the level of infection within individual hex units can be visually appreciated both by the intensity of the color and the height of a bar for each unit. At one point it can readily be appreciated that the spread of free Vir is occurring from the right to the left of this particular view. The cursor is again dragged back and forth to show how the infection spreads till peak value at the height of the acute infection and then slowly recedes as the infection moves into long-term persistence. The same sequence is then analyzed from the standpoint of CTL_Lyt_ (in purple). The video then momentarily pans out to the whole ring once more demonstrating the mobility and versatility of the simulation as it pans round and into the ring whilst pulling up and changing the data boxes. Finally, the simulation once more pans down into the lingual tonsil and observes the spread of Vir.
(83.9 MB AVI)

## Abbreviations

AIM: infectious mononucleosis
CTL: cytotoxic T cell
EBV: Epstein–Barr virus
HEV: high endothelial venules
IRVE: information-rich virtual environment
